# Association between area-level walkability and glycated haemoglobin: a Portuguese population-based study

**DOI:** 10.1186/s12889-024-18627-2

**Published:** 2024-04-23

**Authors:** Regina Sá, Rita Roquette, Andrea Rebecchi, Judite Matias, Jorge Rocha, Maddalena Buffoli, Stefano Capolongo, Ana Isabel Ribeiro, Baltazar Nunes, Carlos Dias, Mafalda Sousa Uva

**Affiliations:** 1 Unidade de Saúde Pública, Agrupamento de Centros de Saúde (ACES) do Baixo Vouga, Aveiro, Portugal; 2 Unidade de Saúde Pública, Agrupamento de Centros de Saúde (ACES) Algarve I, Faro, Portugal; 3 Departamento de Epidemiologia, Instituto Nacional de Saúde Doutor Ricardo Jorge (INSA), Lisboa, Portugal; 4 Design & Health Lab, Department of Architecture, Built environment and Construction Engineering, Politecnico di Milano, Milan, Italy; 5 Instituto de Geografia e Ordenamento do Território (IGOT), Universidade de Lisboa e Laboratório Associado TERRA, Lisbon, Portugal; 6 Unidade de Investigação em Epidemiologia (EPIUnit), Instituto de Saúde Pública da Universidade do Porto, Porto, Portugal; 7 Departamento de Ciências da Saúde Pública e Forenses e Educação Médica, Faculdade de Medicina, Universidade do Porto, Porto, Portugal; 8 Laboratório para a Investigação Integrativa e Translacional em Saúde Populacional (ITR), Porto, Portugal; 9 Centro de Investigação em Saúde Pública (CISP), Escola Nacional de Saúde Pública (ENSP-NOVA), Lisbon, Portugal; 10 Comprehensive Health Research Center (CHRC), Lisbon, Portugal

**Keywords:** Built environment, Urban planning, Walkability, Diabetes, Glycated haemoglobin

## Abstract

Diabetes poses a substantial disease burden, prompting preventive interventions. Physical inactivity, a major risk factor for type 2 diabetes, can potentially be mitigated by enhancing area-level walkability. Despite this, limited population-based studies have investigated the link between walkability and objective diabetes measures. Our study aims to estimate the association between area-level walkability and individual glycated haemoglobin levels in the Portuguese adult population without the diagnosis of diabetes. Data from the 2011 census and an updated street map were obtained to construct a walkability index based on residential density, land-use mix, and street connectivity. Individual health data were sourced from The National Health Examination Survey (INSEF) 2015, a representative survey of the Portuguese adult population. Gamma regression was employed for estimation of the main associations, revealing that residing in moderately walkable areas significantly reduced average glycated haemoglobin levels (Exp(β) = 0.906; 95% CI: 0.821, 0.999) compared to the least walkable areas. The association was less pronounced and not statistically significant for the third tertile of walkability (Exp(β) = 0.919; 95% CI: 0.822, 1.028). Our findings highlight a nonlinear protective association between walkability and glycated haemoglobin, emphasizing the potential policy implications for urban planning, diabetes prevention, and health promotion.

**Supplementary Information:**

The online version contains supplementary material available at 10.1186/s12889-024-18627-2.

## Background

The burden of diabetes mellitus, which has large economic costs [1], is on the rise globally and is expected to continue increasing over the coming years. In Portugal, the National Health Examination Survey (INSEF) estimated that 9.8% of the adult population had diabetes, in 2015 [2], and an increasing trend of its incidence has been observed [3].

In 2017, type 2 diabetes mellitus (T2DM) affected 6.28% of the world’s population, while Portugal presented one of the highest prevalences in Europe [4]. T2DM is determined by genetic, lifestyle, environmental and socioeconomic factors. On the one hand, risk factors for T2DM include lower education levels, tobacco use, and a higher body mass index [5]. On the other hand, engagement in any type of physical activity and a higher intake of vegetables are considered protective factors [5].

Although physical activity is considered to be one of the main determinants of T2DM, globally, one-fourth of adults do not reach the recommended physical activity levels [6]. In the Portuguese population, only 34.2% of adults exercised regularly in 2015 [7].

Environmental and behavioural risk factors are modifiable through effective state interventions. Targeting modifiable risk factors, such as physical activity, is needed to reverse the trend in T2DM incidence. Understanding the factors that lead to its development is key for public interventions. The environment plays an important role in promoting healthy lifestyles and increasing the availability of opportunities for physical activity through active transport [8–10] and leisure-time walking [11]. Walkability is defined by how friendly an area is to walk and consists of two key aspects: proximity to destinations and connectivity. Proximity is determined by the *(i)* diversity of land use (e.g., offices, housing, commerce, entertainment, services) and *(ii)* density, which refers to the number of people, households or jobs distributed by an area unit [12–14]. Connectivity may be measured by the density of street intersections in a given area [14].

Area-level walkability has previously been shown to impact individual physical activity [14–21] and self-reported T2DM [19, 22]. However, little research has estimated the association of walkability with T2DM using a population-based sample [17, 21] and objective outcome measures, such as glycated haemoglobin (HbA_1c_) [17, 23, 24], which allows us to estimate more valid associations. To our knowledge, none of the studies that have simultaneously considered a population-based sample and an objective measure of diabetes have restricted the analysis to individuals without a previous diagnosis of diabetes either. This restriction allows, on the one hand, to eliminate the mean levels of HbA_1c_ of individuals diagnosed with T2DM that are influenced by disease treatment and control and, on the other hand, to identify factors that may affect mean HbA_1c_ levels before diabetes is diagnosed, permitting to obtain evidence for early action in disease prevention [25].

The aim of this study is to estimate the association between area-level walkability and individual levels of HbA_1c_ in the Portuguese adult population without a previous diagnosis of diabetes.

## Methods

### Study population and sample

We used secondary data collected from the National Health Examination Survey (INSEF), a population based survey representative of the Portuguese adult population that has its methods thoroughly described elsewhere [26]. Our study population was the one from the INSEF 2015 performed in Portugal. INSEF followed a multistage sampling method, typical of surveys with geographical representativeness [26]. Data were collected at primary care centres (PCC) and included a general health questionnaire, physical examination, and blood collection for analysis, where HbA_1c_ was assessed. A total of 4911 participants were obtained from 49 primary sampling units (PSU), with a response rate of 43.9% [27]. The study population included noninstitutionalized adults (25–74 years old) who had lived in Portugal for more than a year in 2015, and who were able to understand the Portuguese language [26]. Participants were required to have their current residency correspond to the PSU they were being selected from. Exclusion criteria for blood collection included the existence of chronic disease or known anaemia that would prevent this procedure [26].

**Fig. 1:**
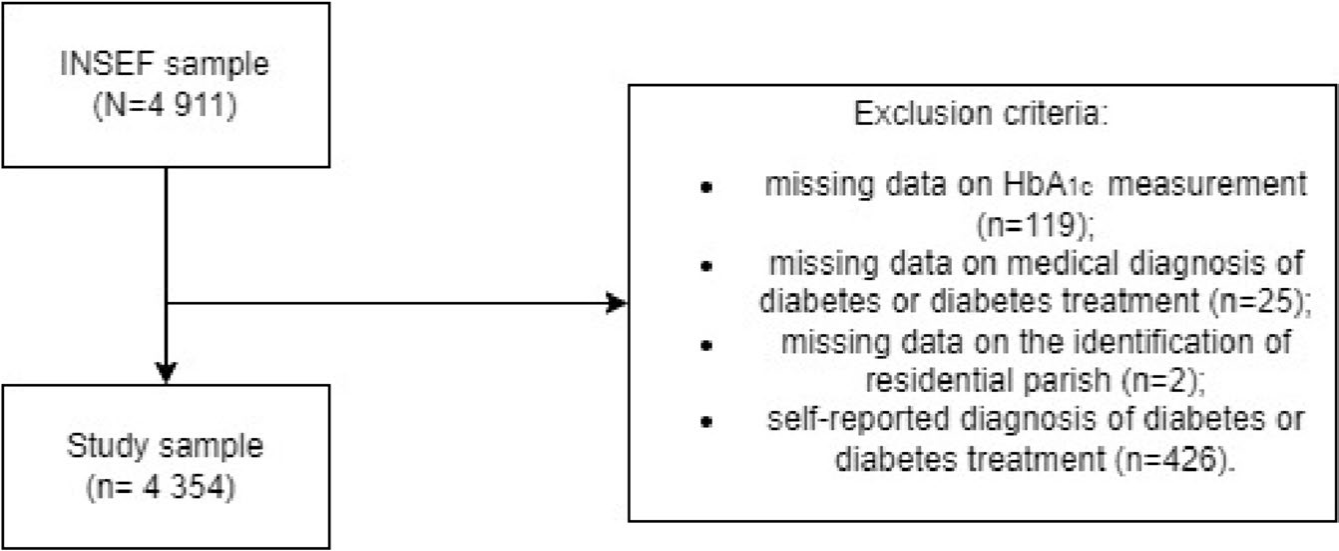
Study sample flow diagram

We conducted a cross-sectional analysis restricted to individuals without a previous diagnosis of diabetes (by self-reported diagnosis made by a medical doctor or by self-reported use of antidiabetic medication in the two weeks before the INSEF interview) (Fig. 1). The exclusion of these individuals allowed *i*) to control for changes in HbA_1c_ that are due to exposures other than those we are studying (e.g., antidiabetic medication) and *ii*) to identify factors that affect HbA_1c_ levels before the diagnosis of diabetes and, therefore, to inform policies aimed at preventing the disease and promoting health. The identification of parishes for each participant was based on their residential address information from INSEF. Observations with missing values for HbA_1c_, residential parish or diabetes treatment or medical diagnosis were excluded from the sample. Some individuals were excluded from the study sample due to more than one criterion.

### Exposure (walkability)

Parishes, the smallest Portuguese administrative division, were used as a scale of analysis of the association between walkability and HbA_1c_. Using smaller scales allows for a more specific estimation, since larger scales could mask important differences within area heterogeneity. In 2011, Portugal consisted of 3429 parishes with an average of 9241 residents and 27km^2^ of area.

The exposure variable was a walkability index at the area level for each Portuguese parish, categorized in tertiles, encompassing the total national territory. The methodology used as a ground framework to measure walkability was a composit index of three indicators [14]: *i*) residential density, the density of classic familiar housing from the census 2011, made available by Statistics Portugal; *ii*) land-use mix, the density of predominantly residential buildings and predominantly non-residential buildings from the census 2011 [28], which did not follow a traditional approach [14] since it was being assessed for a vast geographical area, for which disaggregated data were not available; and *iii*) street connectivity, the density of nodes between three or more walkable streets, from ArcGIS® StreetMap Premium. Finally, the index was calculated by computing the unweighted sum of the three standardized indicators. ArcGIS® version 10.8 was used to calculate the walkability index (WI) and depict its spatial distribution. The rationale for categorizing the walkability index into tertiles was based on established methods in the literature [16, 23] and practical considerations for interpretation. This categorization facilitates the interpretability of our findings and contributes to the robustness of our analysis. More details on the methodology used for this index can be found in (Supplementary Table 1).

### Outcome (HbA_1c_)

Individual-level variables were obtained from INSEF 2015 and included the outcome variable, HbA_1c_. In this study, HbA_1c_ was analysed as a continuous variable. In INSEF, all clinical analyses were performed in different regional laboratories, given the need for results within a maximum period of 24 hours after blood sample collection [26]. All blood sample collection procedures followed the recommendations of the European Health Examination Survey (EHES) [29]. The venous puncture procedure was based on the guidelines of the World Health Organization [30]. The sample collection did not require the participants to fast and the procedure was carried out by a clinical analysis technician or a nurse, using Clipse® [26]. A 2 mL tube (with ethylenediaminetetraacetic acid (EDTA)) of blood sample was used to measure HbA_1c_ levels through high-performance liquid chromatography [26].

INSEF applied corrections to sampling weights to account for non-response in each region, to maintain representativeness and address potential biases [26]. However, it is important to acknowledge that non-response may be associated with individuals less interested in their health status, potentially leading to higher HbA1c values among non-responders. Additionally, exclusion criteria, such as residency in institutions or the inability to participate in interviews, may have led to the exclusion of individuals with more unfavourable health determinants and higher HbA1c levels [26]. These factors were considered in the interpretation of our study results.

### Confounding variables

Individual-level covariates included age (25–29; 30–34; 35–39; 40–44; 45–49; 50–54; 55–59; 60–64; 65–69; 70–74), sex (male and female), education level (no education/1st cycle, 2nd/3rd cycle, secondary and tertiary) and employment status (employed, unemployed, and others without professional activity) [26].

At the area level, the Portuguese version of the European Deprivation Index (EDI) [31], categorized in quintiles, for each parish, was used as a covariate in the regression models, as it previously showed a significant association with HbA_1c_ levels in the Portuguese population [25] and is expected to be associated with area-level walkability.

### Statistical analysis

Descriptive statistics included the characteristics of the sampled individuals, presented as total and relative frequencies, for categorical variables and means and standard deviations (SD) for continuous variables. Comparison of the distribution of HbA_1c_ between groups was performed using Student’s t test or nonparametric tests (i.e., Mann-Whitney or Kruskal-Wallis). The normality of the distribution was assessed using the Shapiro-Wilk test.

All estimates were weighted to account for the different selection probabilities resulting from a study design of complex samples and to correspond to the population distribution in terms of geographic region, age group and gender in 2015 [26]. Individuals with missing data in confounding variables were only excluded for that analysis, this was the case for “unemployment” and “educational level”.

We started by testing multilevel approach to account for the hierarchical structure of our data, where individuals are nested within parishes. Since the levels of HbA_1c_ did not follow a Gaussian distribution, three multilevel generalized linear models were tested with normal, lognormal and gamma distributions [32, 33] using the command meglm from the svy package of Version 15 of Stata®. The selection of the model distribution was based on the smallest Akaike’s information criterion (AIC). We tested the aggregation of the observations regarding the clustering variable (parish) in a multilevel null model. Variance and the number of observations in each parish were analysed to consider a multilevel analysis.

To better measure the association between area-level walkability and HbA_1c_ mean values, a multivariate, single-level analysis was run using a gamma regression with a log link. The selection of variables to properly adjust for confounding was performed according to a literature review, and using a directed acyclic graph (DAG), built in DAGitty v3 (Supplementary Fig. 1), and the backdoor method. Four models were tested in a forward fashion, using: *i*) the continuous outcome variable (mean HbA_1c_) and the exposure variable (area-level walkability tertiles); *ii*) the previous variables and demographic variables (i.e., sex and age group) [16, 17, 23, 24, 34–36]; *iii*) the previous variables and socioeconomic individual-level variables (i.e., education and employment) [16, 17, 23, 24, 34]; and *iv*) all previous variables adding the area-level socioeconomic deprivation index [16, 17, 24, 34].

We compared the mean values of HbA_1c_ between classes of the exposure variable (walkability index), using the least walkable category as a reference. Exponentiated beta coefficients and respective 95% Confidence intervals (95% CI) are presented. The interpretation of the coefficients is as follows: (exp(β)-1)×100 mean percentage of change in HbA_1c_. The 95% CI of the coefficients and the standard error of the main association were used to assess the models.

All statistical analyses were performed in Stata® version 15, and the statistical confidence level was set at 95%.

## Results

### Characteristics of the study sample

A total of 4352 individuals, from 490 parishes (11.49% of the total number of Portuguese parishes in 2011), were included in the analysis. On average, each parish had 9 individuals, ranging in age from 1 to 71. This sample (Table 1) had a higher proportion of females (54.87%) and individuals aged between 40 and 44 years (13.56%). Regarding socioeconomic aspects, individuals with lower education levels (secondary school or lower) (81.67%) and employed individuals (63.76%) were more common. Finally, a greater number of participants (28.75%) lived in parishes with medium-high levels of socioeconomic deprivation (4th quintile).

The descriptive analysis of the sample according to the tertiles of walkability showed significant differences for: *(i)* the level of education (increasing levels of walkability were associated with an increase in education); and *(ii)* the socioeconomic deprivation (a higher proportion of participants living in the least and most walkable areas were in the 4th quintile of EDI (medium-high levels of socioeconomic deprivation) (35.66% and 33.38%), but those living in medium-walkable areas were mainly in the 2nd quintile of EDI (medium-low levels of socioeconomic deprivation) (25.17%)).

**Table 1:** Characteristics of INSEF 2015 participants without a previous diagnosis of diabetes and presenting data for parish and HbA_1c_ (*N* = 4 352)

Characteristics	n	%	Least Walkable n (%)	Medium-walkable n (%)	Most Walkable n (%)	*P* value
**Sex**						
Male	1964	45.13	341 (46.58%)	442 (43.46%)	1181 (45.13%)	0.400
Female	2388	54.87	391 (53.41%)	575 (56.54)	1422 (54.63%)	
**Age groups (years)**						
25–29	322	7.40	76 (10.38%)	80 (7.87%)	166 (6.38%)	0.016
30–34	371	8.52	53 (7.24%)	101 (9.93%)	217 (8.34%)	
35–39	518	11.90	79 (10.79%)	130 (12.78%)	309 (11.87%)	
40–44	590	13.56	106 (14.48%)	135 (13.27%)	349 (13.41%)	
45–49	526	12.09	74 (10.11%)	128 (12.59%)	324 (12.45%)	
50–54	558	12.82	88 (12.02%)	114 (11.21%)	356 (13.68%)	
55–59	443	10.18	79 (9.97%)	96 (9.44%)	274 (10.53%)	
60–64	441	10.13	68 (9.29%)	111 (10.91%)	262 (10.07%)	
65–69	331	7.61	68 (9.29%)	64 (6.29%)	199 (7.65%)	
70–74	252	5.79	47 (6.42%)	58 (5.70%)	147 (5.65%)	
**Education**						
No education/1st cycle	1209	27.78	241 (32.92%)	311 (30.58%)	657 (25.24%)	< 0.001
2nd/3rd cycle	1473	33.85	284 (38.80%)	343 (33.73%)	846 (32.50%)	
Secondary	872	20.04	121 (16.53%)	217 (21.34%)	534 (20.51%)	
Tertiary	794	18.24	84 (11.48%)	145 (14.26%)	565 (21.71%)	
**Employment**						
Employed	2775	63.76	447 (66.22%)	650 (70.65%)	1,678 (68.77%)	0.118
Unemployed	488	11.21	82 (12.15%)	100 (10.87%)	306 (12.54%)	
Others without professional activity (retirees. housewives. students and disabled)	1086	24.95	203 (27.73%)	267 (26.25%)	616 (23.67%)	
**Area socioeconomic deprivation (quintiles)**						
1st (least deprived)	428	9.83	33 (4.51%)	255 (25.07%)	140 (5.38%)	< 0.001
2nd	760	17.46	150 (20.49%)	256 (25.17%)	354 (13.60%)	
3rd	791	18.18	72 (9.84%)	238 (23.40%)	481 (18.48%)	
4th	1251	28.75	261 (35.66%)	121 (11.90%)	869 (33.38%)	
5th (most deprived)	1122	25.78	216 (29.51%)	147 (14.45%)	759 (29.16%)	

Proportions of the total number of observations are read in the row. Proportions for each walkability tertile are read in the column

*P* value refers to the Chi2 test for differences in the distribution of the variables according to the walkability tertile. No cell presented expected values bellow 5

In the study sample, the mean HbA_1c_ level was 5.35%, with a range of 3.7–14.1%. The median was 5.3% (25th percentile = 5.1%; 75th percentile = 5.6%). When analysing HbA_1c_ regarding sample characteristics, female sex, younger age, higher education levels and employment shoed lower values (Supplementary Table 2). No significant association was found between the quintiles of socioeconomic area-level deprivation and the mean levels of HbA_1c_. The proportion of individuals with a mean HbA_1c_ level above 6.5%, the currently recommended threshold for the diagnosis of diabetes [37], was 1.24%, which corresponds to underdiagnosis.

### Area-level walkability

**Fig. 2:**
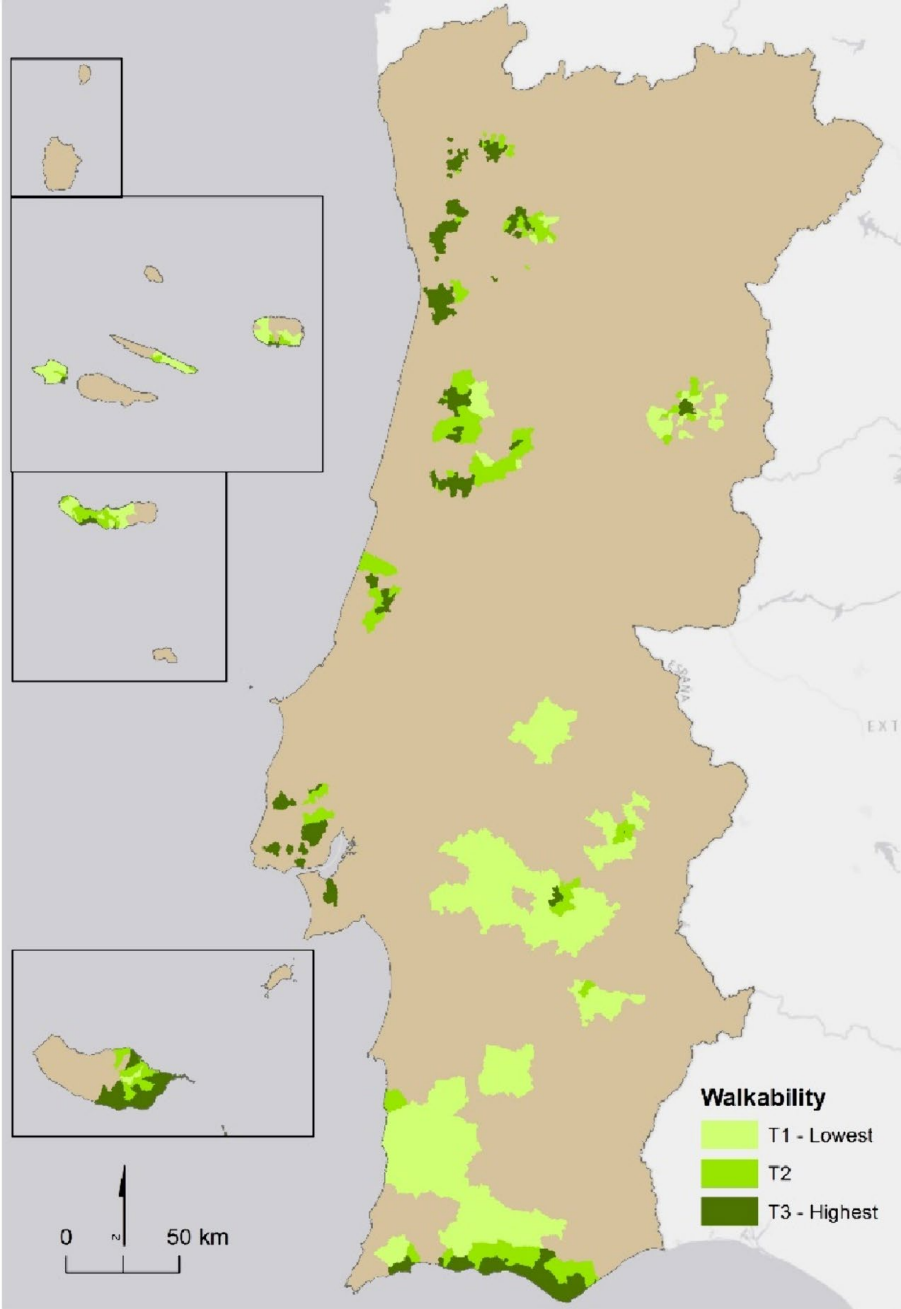
Map of Portugal referring to the tertiles of the walkability index in the parishes considered in INSEF 2015

The spatial distribution of the walkability index among INSEF 2015 parishes is shown in Fig. 2. From the study sample, 16.82% of participants resided in least walkable areas (1st tertile), 23.37% in medium-walkable areas (2nd tertile), and 59.81% in most walkable areas (3rd tertile). More participants lived in areas of higher walkability due to the higher residential density of those parishes, therefore accommodating a higher number of individuals.

### Association between area-level walkability and HbA_1c_ levels

The gamma distribution was considered for the HbA_1c_ levels since it showed the lowest value of AIC. The null model, using only the outcome (HbA_1c_ levels) and the clustering variable (parish), showed a low variance (near zero); therefore, random effects were considered statistically nonsignificant. Additionally, the number of observations in each parish was too low to run a multilevel analysis (Supplementary Table 3).

Single-level multivariate gamma regression results are presented in Table 2. The estimate of the ratio of mean HbA_1c_ between walkability levels, in model 1 did not show statistically significant associations, although the coefficients were all below one [Exp(β) = 0.894(95% CI 0.797, 1.001) for medium-walkable and 0.898 (95% CI 0.795, 1.013) for most walkable], pointing to a protective association. Model 2, adding demographic variables, showed higher yet coefficients for the main association, with 0.904 (95% CI 0.815, 1.002) for medium walkable, and 0.909 (95% CI 0.815, 1.015) for most walkable, pointing to a reduction in the protective association but an increase in precision. Similarly, in model 3, with the addition of socioeconomic variables (educational level and employment), the coefficients of the main association increased again, with 0.905 (95% CI 0.817, 1.003) for the 2nd tertile of walkability and 0.917(95% CI 0.821, 1.025) for the 3rd tertile of walkability. Finally, in model 4, with the addition of area socioeconomic deprivation, the main association achieved statistical significance for the 2nd tertile of walkability showing an Exp(β) = 0.906(95% CI: 0.821, 0.999). The 3rd tertile of walkability, showed an Exp(β) = 0.919(95% CI: 0.822, 1.028). In the fully adjusted model (4), when compared to least walkable areas, living in medium-walkable areas reduced, in a statistically significant way, the mean levels of HbA_1c_ in 9.4%, and living in most walkable areas reduced the mean levels of HbA_1c_ in 8.1%. Since for most walkable areas, the estimated 95% CI included the value 1, the association was not considered statistically significant; however, it is very close to being significant.

**Table 2:** Results of single-level gamma regression of the association between walkability at the area level and mean HbA_1c_ (*n* = 4352)

Variables	Categories	n	Mean HbA_1c_ (%)	Model 1	Model 2	Model 3	Model 4
				Exp(β) (95% CI)	Exp(β) (95% CI)	Exp(β) (95% CI)	Exp(β) (95% CI)
Walkability (tertiles)	^R^Least walkable	732	5.45	-	-	-	-
	Medium walkable	1017	5.33	0.894(0.797–1.001)	0.904(0.815–1.002)	0,905(0,817–1,003)	0,906(0,821–0,999)
	Most walkable	2603	5.34	0.898(0.795–1.013)	0.909(0.815–1.015)	0,917(0,821–1,025)	0,919(0,822–1,028)
Sex	Female	2388	5.34		0.973(0.939–1.007)	0,979(0,949–1,011)	0,98 (0,95 − 1,011)
	^R^Male	1964	5.35		-	-	-
Age group	25–29	322	5.09		0.591(0.539–0.649)	0,597(0,542–0,658)	0,595(0,540–0,656)
	30–34	371	5.18		0.647(0.599–0.698)	0,651(0,594–0,713)	0,648(0,590–0,712)
	35–39	518	5.18		0.649(0.581–0.726)	0,651(0,583–0,727)	0,650(0,581–0,727)
	40–44	590	5.26		0.7(0.629–0.779)	0,698(0,619–0,788)	0,697(0,618–0,787)
	45–49	526	5.37		0.787(0.678–0.914)	0,780(0,679–0,896)	0,778(0,678–0,893)
	50–54	558	5.40		0.811(0.733–0.897)	0,801(0,72 − 0,891)	0,800(0,719–0,890)
	55–59	443	5.50		0.891(0.778–1.021)	0,881(0,755–1,029)	0,878(0,752–1,026)
	60–64	441	5.54		0.935(0.844–1.035)	0,927(0,83 − 1,036)	0,926(0,830–1,035)
	65–69	331	5.52		0.909(0.83–0.995)	0,91(0,831–0,997)	0,908(0,828–0,996)
	^R^70-74	252	5.61		-	-	-
Educational level	^R^No education/ basic 1st cycle	1209	5.5			-	-
	Basic 2nd/3rd cycle	1473	5.35			0,985(0,922–1,051)	0,984(0,922–1,05)
	Secondary	872	5.26			0,948(0,894–1,005)	0,946(0,892–1,003)
	Higher	794	5.23			0,933(0,875–0,995)	0,932(0,873–0,994)
Employment	Employed	2775	5.29			1,026(0,959–1,098)	1,027(0,96 − 1,099)
	Other without professional activity	1086	5.5			0,994(0,915–1,081)	0,994(0,916–1,078)
	^R^Unemployed	488	5.31			-	-
Area socioeconomic deprivation (quintiles)	1st (least deprived)	428	5.34				0,983(0,914–1,058)
	2nd	760	5.36				1,02(0,946–1,099)
	3rd	791	5.35				0,984(0,92 − 1,052)
	4th	1251	5.33				0,971(0,907–1,04)
	^R^5th (most deprived)	1122	5.34				-

## Discussion

In this study, we used a gamma regression to estimate the association of area-level walkability with mean HbA_1c_ levels adjusted for confounding factors. Findings from this study revealed that individuals living in medium and most walkable areas (2nd and 3rd tertiles) were found to have lower mean levels of HbA_1c_ than those living in least walkable areas (1st tertile). Although none of the estimates obtained by comparing most walkable (3rd tertile) with least walkable areas (1st tertile) achieved statistical significance, coefficients remained lower than 1, always suggesting a protective association. In the final adjusted model, living in a medium-walkable parish significantly reduced the mean levels of HbA_1c_ by 9.4% (95% CI 0.1%, 17.9%) compared to living in the least walkable parish. Although not significant, most walkable areas presented a reduction of 8.1% (95% CI -2.8%, 17.8%) in the mean HbA_1c_ levels compared to living in the least walkable area.

A previous meta-analysis estimated that most walkable areas reduced the risk of T2DM, with a pooled relative risk of 0.79 (95% CI 0.72, 0.87) [10]. Although the direction of the association is the same as that found in our study (walkability being a protective factor for T2DM), the studies included in this meta-analysis considered the T2DM diagnosis and not the mean levels of HbA_1c_, and they did not restrict the sample to individuals without a previous diagnosis of diabetes. A study published by Fazli et al. (2020), which performed the same restrictions as we did, found the prediabetes incidence to be 17% higher among participants living in the least versus most walkable neighbourhoods after adjustment for confounding factors [24]. Nevertheless, this estimate is also not comparable with ours since they are not comparing mean HbA_1c_ levels, but prediabetes as a dichotomic variable. Additionally, their study used a nonrepresentative sample obtained from a laboratory database. This method can lead to selection bias because healthier people are less likely to use healthcare services and have laboratory exams undertaken, leading to an overestimation of the effect of walkability on HbA_1c_ levels. Another recent study that used a similar methodology, found no statistically significant association between objectively measured walkability and change in HbA_1c_ levels in the fully adjusted model, with coefficients of -1.12 (95% CI -2.26, 0.03) and − 0.45 (95% CI -0.162, 0.72) in the 2nd and 3rd tertiles, respectively [16]. This was a cohort study from The Netherlands, based on registries from the care centre, and therefore prone to selection biases too. Finally, a French study with a similar methodology also found no association between the walkability index and mean differences in HbA_1c_ [23]. However, a nonsignificant increase in mean HbA_1c_ levels was observed to be associated with high walkability in the main model. Although this is a population-based study like ours, consisting of a cross-sectional survey, it only restricted its sample to participants not reporting the use of diabetes medication in the sensitivity analysis. Using the same sample restrictions, Hajna et al. were also not able to find associations between neighbourhood environment and HbA_1c_ [38].

In the present study, restricting the sample to individuals without a previous diagnosis of diabetes excludes those on glucose-lowering interventions, which is important to understand the true relationship between area-level walkability and HbA_1c_ but could also reduce the strength of the association found. Individuals in the lower tertiles of walkability could present higher HbA_1c_ levels but were excluded due to diagnosis, leading to a selective selection bias. This bias could lead to an underestimation of the association found here.

We observed that mean levels of HbA_1c_ increased significantly from the medium to the most walkable areas. However, the association between area-level walkability and HbA_1c_ mean levels may not be strong enough to reflect the statistical associations. Considering the indicators that we used to estimate the walkability index (residential density, land-use mix and street connectivity), we point out three other explanations for such phenomena.

The first explanation would be the burden associated with urban areas, which score better in the index but also present higher frequencies of some environmental risk factors for T2DM, such as: *i*) the food environment [39]; *ii*) the socioeconomic environment [39]; and *iii*) the physical environment (e.g., higher levels of air pollution and noise and lower green space availability) [10, 40]. Such a phenomenon, with opposite effects on walkability, could underestimate the association reported here. In our study, we were able to decrease the confounding bias of the second by adjusting for area socioeconomic deprivation, which led to an increase in the precision of the association of interest in model 5. Future studies should consider including the food environment (e.g., availability of fast-food restaurants) and physical environment (e.g., PM_10_, NO_2_, green space availability, or noise levels) to test this hypothesis.

The second explanation could be the potentially negative effect of too high area-level walkability. Counterintuitively, hyper-proximity of destinations could reduce the cumulative periods of physical activity. Actually, a cohort study of diabetic people who considered neighbourhood self-selection, developed in The Netherlands, found that higher neighbourhood walkability was associated with lower physical activity [16]. Another Canadian study observed associations between higher neighbourhood walkability and lower obesity and decreased incidence of diabetes, but not with physical activity [41]. On the one hand, this justifies the importance of clarifying the levels of physical activity in areas of extremely high density and land-use mix. On the other hand, this supports the potential importance of other pathways in the causal association than physical activity, since indirect mechanisms could mediate this association [16, 34, 41].

Finally, the third explanation is based on the hypothesis that areas in the 3rd tertile of walkability may present very different contexts, which include city centres (e.g., Porto and Lisbon) and suburban areas (e.g., Vila Nova de Gaia and Amadora). Although city centres are more favourable to active transport, in the periphery there will be a greater tendency towards commuting and, therefore, the use of passive transport (e.g., trains or cars) [42]. In fact, suburban areas have been recently identified as the worst of the two worlds, with the deleterious effects of urban and rural settings. A study from the United Kingdom found a U-shaped association between residential density and physical activity, where suburban areas (1800–3200 units per km^2^) presented the worst health outcomes [43]. Future studies should consider the double burden of suburban areas.

Our study has some limitations. First, in the INSEF sample, the number of individuals per parish was small, and several authors report a minimum of 20–30 individuals per group to justify running a multilevel model [44, 45]. Additionally, the variance of the random effects (referring to the area level) was very close to zero, meaning that the between-group differences for each parish with regard to HbA_1c_ are not relevant. However, running a single-level analysis should not affect the answer to the research question in this case, since there is no interest in identifying independent units (e.g., parishes with better or worse HbA_1c_ levels). Other studies with similar sampling reported single-level models in their analysis [19, 46]. Second, the place of residence of INSEF participants was the place in which they lived for at least 12 months. Thus, for individuals who recently changed from one parish to another, the influence of area walkability on the mean levels of HbA_1c_ was unclear. Third, in Portugal, parishes vary in population size and area and may contain subgroups with diverse degrees of walkability. Such misclassification bias could be reduced if an area-level variable with smaller geographical units was considered, such as neighbourhoods. The number of INSEF participants in each would be very small, leading to a lack of statistical power and increasing random errors. Moreover, many policy interventions are implemented at the parish level, making this analysis relevant for assessing intervention impacts and tailoring strategies to local needs. Fourth, observational aspects of the urban environment (e.g., safety and aesthetics) were not considered in this study [47]. These aspects have previously been shown to have a positive influence on diabetes [48] and could underestimate the associations found here. Fifth, the least walkable areas may foster individuals with higher HbA_1c_ levels because of reduced access to health promotion and disease prevention services. Although this aspect may contribute to the reported associations being stronger, only 1.24% of the individuals included in our analysis had HbA1c levels above the cut-off for diabetes diagnosis. Furthermore, although regional disparities are present in Portugal, primary care is universal and free in all territory [49]. Sixth, the exclusion criteria employed by INSEF 2015 may have resulted in the leaving out individuals with poorer health determinants and elevated HbA1c levels, potentially exerting an influence on our findings and attenuating the strength of our main association. Seventh, factors other than glycemia may influence HbA1c levels, these include changes in erythropoiesis, haemoglobinopathies, status that increase or decrease glycation, erythrocyte destruction, and factors that may influence the assays [37], That said, the use of HbA1c is still the most widely accepted method to diagnose DM.

The major strength of this study is the fact that it combined, a population-based sample, representative of the Portuguese adult population, and an objective measure of diabetes (HbA_1c_). Instead of self-reporting, by using an objective exposure variable [17, 21, 24, 41] and an objective outcome measure [17, 23, 24, 50], in addition to using covariates such as the Portuguese version of the European Deprivation Index, this study is easily replicable in time and space and allows comparison between countries. Furthermore, the restriction of analysis to individuals without a previous diagnosis of diabetes may lead to knowledge that is helpful for disease prevention [24, 50]. Thus, our study may be useful to formulate preventive solutions that act sooner in the natural history of disease, preventing its installation. Additionally, this work was able to develop a walkability index for the entirety of Portugal, which did not yet exist and could serve as a basis for other future investigations.

## Conclusion

Our findings suggest a nonlinear protective effect of walkability on T2DM. This study highlights the importance of considering the aspects of walkability in urban planning, with the goal of preventing diabetes and promoting health through environmental policies. In addition, this walkability index can be used as an informative tool for policy makers when developing urban plans.

### Supplementary Information

**Supplementary Material 1.**

## Data Availability

The datasets generated and analysed during the current study are not publicly available due to intellectual property reasons but are available from the corresponding author on reasonable request. Publicly archived datasets from the Census used in this study can be found in Statistics Portugal website https://www.ine.pt/. Datasets from the INSEF 2015 used in this study are property of the National Institute of Health Doutor Ricardo Jorge (INSA) and can be requested to the entity, with restricted conditions for access.

## Abbreviations

INSEF: National Health Examination Survey
T2DM: Type 2 Diabetes Mellitus
HbA_1c_: Glycated haemoglobin
PCC: Primary Care Centres
PSU: Primary Sampling Units
EHES: European Health Examination Survey
EDTA: Ethylenediaminetetraacetic Acid
EDI: European Deprivation Index
AIC: Akaike’s information criterion
DAG: Directed Acyclic Graph
