# Supplementary material for: Association between area-level walkability and glycated haemoglobin: a Portuguese population-based study
Source: BMC Public Health. 2024 Apr 23;24:1116. doi: 10.1186/s12889-024-18627-2 (PMC11036776; doi:10.1186/s12889-024-18627-2)
Supplement: Supplementary file 1 — Supplementary Material 1. [file 12889_2024_18627_MOESM1_ESM.docx]

**Supplementary material**

**Figure S1** Graph for model’s variable selection.


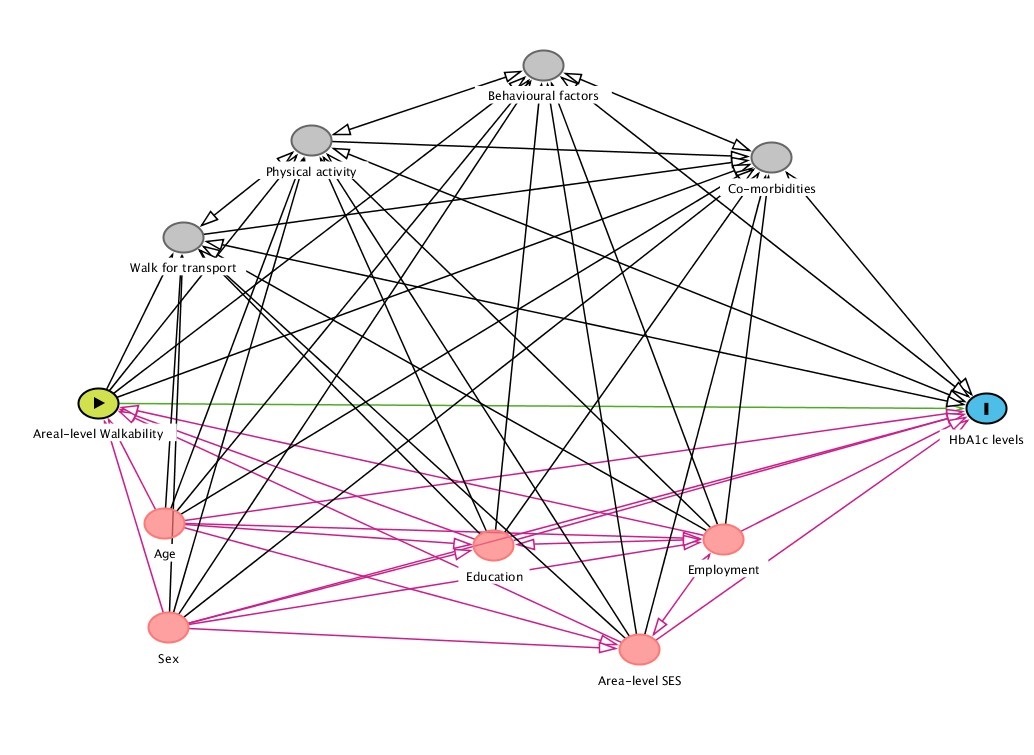


Legend: Green – ancestors, pink – confounder, grey –mediators.

**Table S1** Indicators, data sources, numerator, denominator, and standardization methods used for the *Walkability Index*

| **Indicator / Index** | **Data source** | **Numerator** | **Denominator** | **Standardization method** |
| --- | --- | --- | --- | --- |
| **Residential Density**  **(RD)** | 2011 Census (INE) | Nº8 of classic family dwellings | Parish area (km2) | $\frac{RD-RDmin}{RDmax-RDmin}$ |
| **Land use diversity**  **(LUD)** | 2011 Census (INE) | Sum of predominantly residential buildings and predominantly non-residential buildings (excludes exclusively residential buildings) | Parish area (km2) | $\frac{LUD-LUDmin}{LUDmax-LUDmin}$ |
| **Connectivity of streets (CS)** | ESRI® | Nodes between three or more walkable roads | Parish area (km2) | $\frac{CS-CSmin}{CSmax-CSmin}$ |
| **Walkability Index (WI)** | $WI=RDst+LUDst+CSst$ | | | |

**Table S2** Distribution of the glycosylated haemoglobin values (%) according to the sample characteristics

|  | Classes | Average | Standard Error | IC95 | | *P-value* |
| --- | --- | --- | --- | --- | --- | --- |
| Sex | Female | 5.34 | 0.02 | 5.30 | 5.38 | 0.038* |
|  | Male | 5.35 | 0.02 | 5.31 | 5.39 |  |
| Age group | 25-29 | 5.09 | 0.03 | 5.03 | 5.14 | <0.001 |
|  | 30-34 | 5.18 | 0.04 | 5.09 | 5.26 |  |
|  | 35-39 | 5.18 | 0.03 | 5.13 | 5.24 |  |
|  | 40-44 | 5.26 | 0.03 | 5.20 | 5.31 |  |
|  | 45-49 | 5.37 | 0.05 | 5.28 | 5.47 |  |
|  | 50-54 | 5.40 | 0.02 | 5.36 | 5.44 |  |
|  | 55-59 | 5.50 | 0.07 | 5.35 | 5.64 |  |
|  | 60-64 | 5.54 | 0.03 | 5.48 | 5.61 |  |
|  | 65-69 | 5.52 | 0.04 | 5.44 | 5.59 |  |
|  | 70-74 | 5.61 | 0.05 | 5.51 | 5.72 |  |
| Level of education | No education/ 1st basic cycle | 5.50 | 0.04 | 5.43 | 5.58 | <0.001 |
|  | 2nd/3rd basic cycle | 5.35 | 0.02 | 5.31 | 5.39 |  |
|  | Secondary | 5.26 | 0.02 | 5.22 | 5.30 |  |
|  | Superior | 5.23 | 0.03 | 5.17 | 5.28 |  |
| Employment | Employee | 5.29 | 0.02 | 5.25 | 5.32 | <0.001 |
|  | Unemployed | 5.31 | 0.03 | 5.24 | 5.38 |  |
|  | Another without professional activity | 5.50 | 0.04 | 5.43 | 5.58 |  |
| Area socio-economic deprivation index (quintiles) | 1 | 5.34 | 0.03 | 5.27 | 5.40 | 0.102 |
|  | 2 | 5.36 | 0.04 | 5.28 | 5.44 |  |
|  | 3 | 5.35 | 0.03 | 5.29 | 5.41 |  |
|  | 4 | 5.33 | 0.02 | 5.28 | 5.38 |  |
|  | 5 | 5.34 | 0.03 | 5.29 | 5.40 |  |
| Area walkability (tertiles) | 1 | 5.45 | 0.06 | 5.32 | 5.57 | 0.018 |
|  | 2 | 5.33 | 0.02 | 5.28 | 5.38 |  |
|  | 3 | 5.34 | 0.02 | 5.30 | 5.37 |  |
| *P-value* referring to the difference test (Kruskal-Wallis) between the various groups regarding the distribution of glycosylated hemoglobin. The null hypothesis is that there is no difference in the medians between the groups. *Mann Whitney: Null hypothesis is that there are no differences in means between groups. | | | | | | |

**Table S3** Characteristics of the null model using a multilevel analysis

| HBA1C | exp(b) | P>\|z\| | 95% CI | |
| --- | --- | --- | --- | --- |
| cons | 5,359 | 0 | 5,340 | 5,379 |
| logs | -2,526 |  | -2,548 | -2,505 |
| parish (variance) | 0,001 |  | 0,000 | 0,001 |
| *Mixed-effects GLM* | |  |  |  |
| gamma |  |  |  |  |
| Log link |  |  |  |  |
| Group variable: parish | |  |  |  |
| Number of groups: 490 | |  |  |  |
| Observations per group: |  |  |  |  |
| minimum=1 |  |  |  |  |
| average=8,9 |  |  |  |  |
| maximum=71 |  |  |  |  |
| *Log likelihood* = -2585.8883 | |  |  |  |
